# Interactive Medical Image Labeling Tool to Construct a Robust Convolutional Neural Network Training Data Set: Development and Validation Study

**DOI:** 10.2196/37284

**Published:** 2022-08-22

**Authors:** David Reifs, Ramon Reig-Bolaño, Marta Casals, Sergi Grau-Carrion

**Affiliations:** 1 Digital Care Research Group, Centre for Health and Social Care Universitat of Vic-Central University of Catalonia Vic Spain; 2 Hospital Santa Creu de Vic Vic Spain

**Keywords:** wound assessment, pressure ulcers, wound tissue classification, labeling, machine learning

## Abstract

**Background:**

Skin ulcers are an important cause of morbidity and mortality everywhere in the world and occur due to several causes, including diabetes mellitus, peripheral neuropathy, immobility, pressure, arteriosclerosis, infections, and venous insufficiency. Ulcers are lesions that fail to undergo an orderly healing process and produce functional and anatomical integrity in the expected time. In most cases, the methods of analysis used nowadays are rudimentary, which leads to errors and the use of invasive and uncomfortable techniques on patients. There are many studies that use a convolutional neural network to classify the different tissues in a wound. To obtain good results, the network must be trained with a correctly labeled data set by an expert in wound assessment. Typically, it is difficult to label pixel by pixel using a professional photo editor software, as this requires extensive time and effort from a health professional.

**Objective:**

The aim of this paper is to implement a new, fast, and accurate method of labeling wound samples for training a neural network to classify different tissues.

**Methods:**

We developed a support tool and evaluated its accuracy and reliability. We also compared the support tool classification with a digital gold standard (labeling the data with an image editing software).

**Results:**

The obtained comparison between the gold standard and the proposed method was 0.9789 for background, 0.9842 for intact skin, 0.8426 for granulation tissue, 0.9309 for slough, and 0.9871 for necrotic. The obtained speed on average was 2.6, compared to that of an advanced image editing user.

**Conclusions:**

This method increases tagging speed on average compared to an advanced image editing user. This increase is greater with untrained users. The samples obtained with the new system are indistinguishable from the samples made with the gold standard.

## Introduction

Skin ulcers are an important cause of morbidity and mortality everywhere in the world [1] and occur due to several causes, including diabetes mellitus, peripheral neuropathy, immobility, pressure, arteriosclerosis, infections, and venous insufficiency. Ulcers are lesions that fail to undergo an orderly healing process and produce functional and anatomical integrity in the expected time (4 weeks to 3 months) [2]. This is usually due to an underlying pathology that prevents or delays healing. Ulcers have a major impact on the patient's life, causing a reduction in the quality of life in physical, emotional [3], and social dimensions. Several contributing and confounding factors are associated with both the cause and maintenance of ulcers. In addition, care of these wounds requires the expenditure of human and material resources and generates a great economic impact [4]. For these reasons, complex wounds such as ulcers are considered a major global problem.

In most cases, the methods of analysis used nowadays are rudimentary, which leads to errors and the use of invasive and uncomfortable techniques for patients. It is extremely difficult to monitor [5] the evolution of the wound based on the healing process as no data are stored or classified efficiently. Literature covering different algorithms focused on the detection and characterization of wounds is limited and mainly based on the capture of size and depth of the wounds [6,7]. There are many studies that use a convolutional neural network (CNN) to classify the different tissues in a wound [8-11]. However, the process of labeling the images for the training of a CNN in a supervised algorithm is hard work and requires extensive time and effort by a health professional.

In current CNN training models, the labeling of the data set samples is a critical and important phase. In pretrained classification networks, images have been labeled using polygonal contour tools that help detect objects, parts of a body, animals, and so on [12]. For tissue classification, more detailed labeling is required. A wound expert user will have to label the samples, typically using a professional photo editing software. Using the editing tools, this user will paint the different tissues of the wound with predetermined colors (eg, granulated in red, slough in yellow, necrotic in black, and intact skin in blue), pixel by pixel. At the end of the process, 2 files are obtained—1 with the original image and 1 modified with labels drawn with the editing software.

The main goal of this work is to propose an interactive tool for labeling wound samples used for training a CNN to classify different tissues. With this interactive tool, the labeling process is faster, more efficient, and more accurate than with the current manual methods.

## Methods

### Materials

The collection of the necessary data for labeling was made with a mobile app that uses a standard camera—in our case, a Samsung Galaxy S10 tablet. The data were collected in a health center by health care professionals.

### Ethics Approval

The clinical protocol has been approved by the CEIC of the Hospital General de Vic (2019093/PR224).

### Proposal

**Figure 1:**
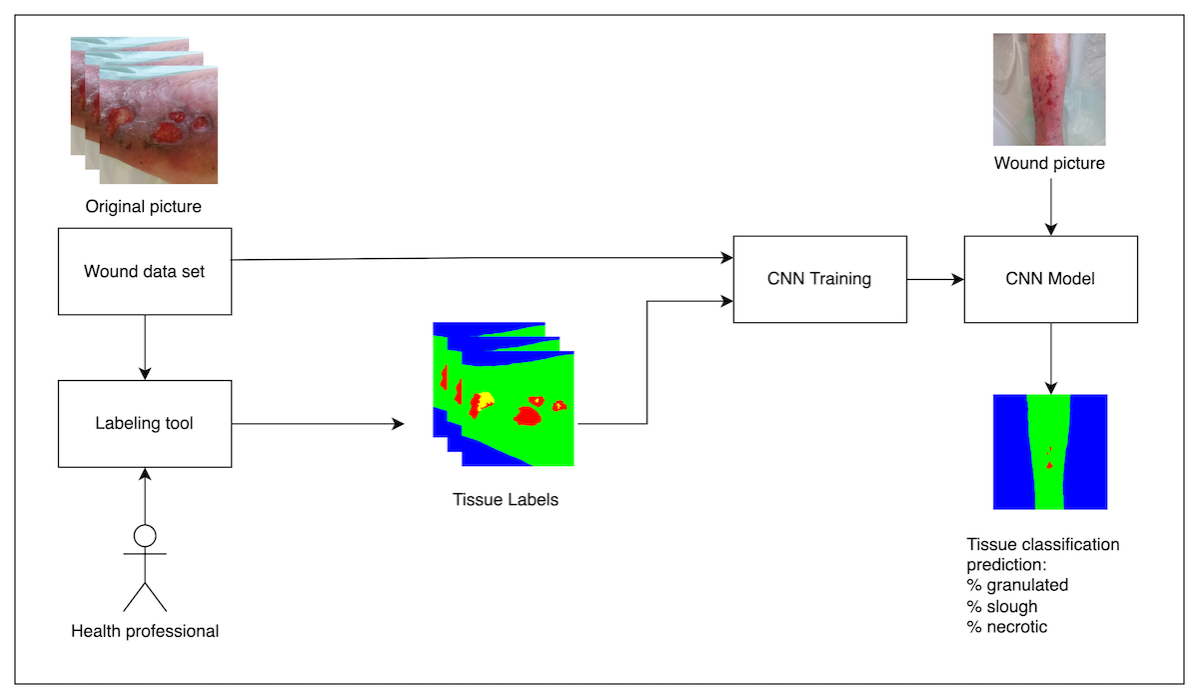
Generic overview of convolutional neural network (CNN) labeling, training, and inference process.

**Figure 2:**
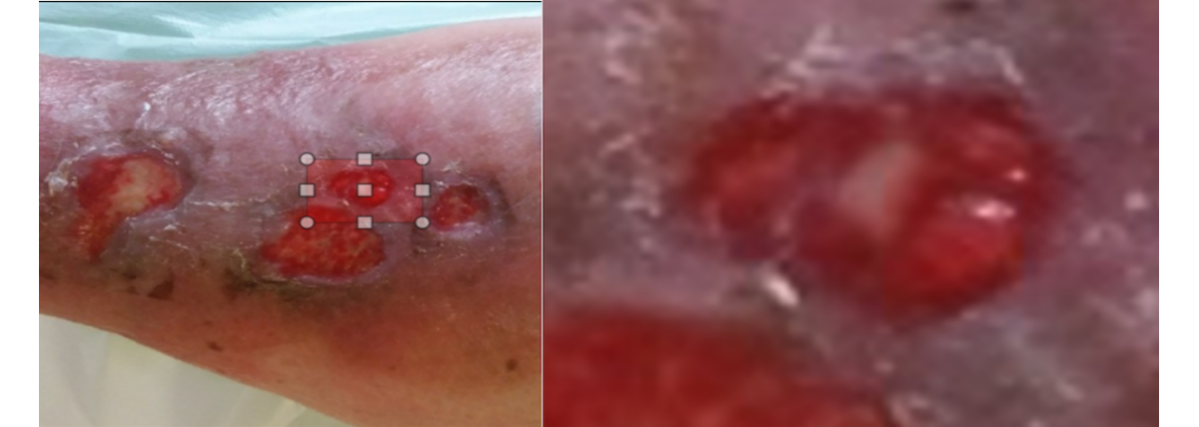
Region selection to apply zoom (left) and the region zoomed (right).

**Figure 3:**
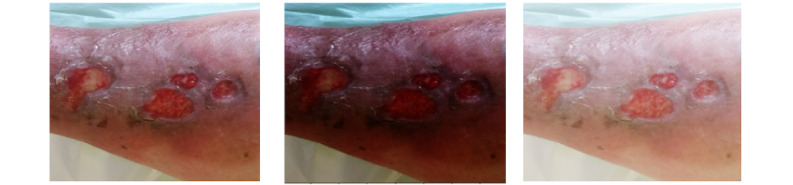
The luminosity of the image can be modified by applying gamma correction. From left to right: original image, gamma value=0.5, and gamma value=2.

A proposed labeling tool is developed and presented in this study. The results of this application are used for training the CNN model (see the complete working framework in Figure 1). This tool is based on an image editor tool and allows for standard image editing actions such as zoom (Figure 2) and gamma correction (Figure 3). It uses computer vision techniques for tagging and labeling each tissue. 

The interactive labeling tool can be divided into 2 working stages. In the first stage, the user can choose the part of the image of interest, using the mouse on the original image to define the region of interest (region to label). At the same time, the user can change the image parameters and hyperparameters of the automatic segmentation methods included in the tool.

**Figure 4:**
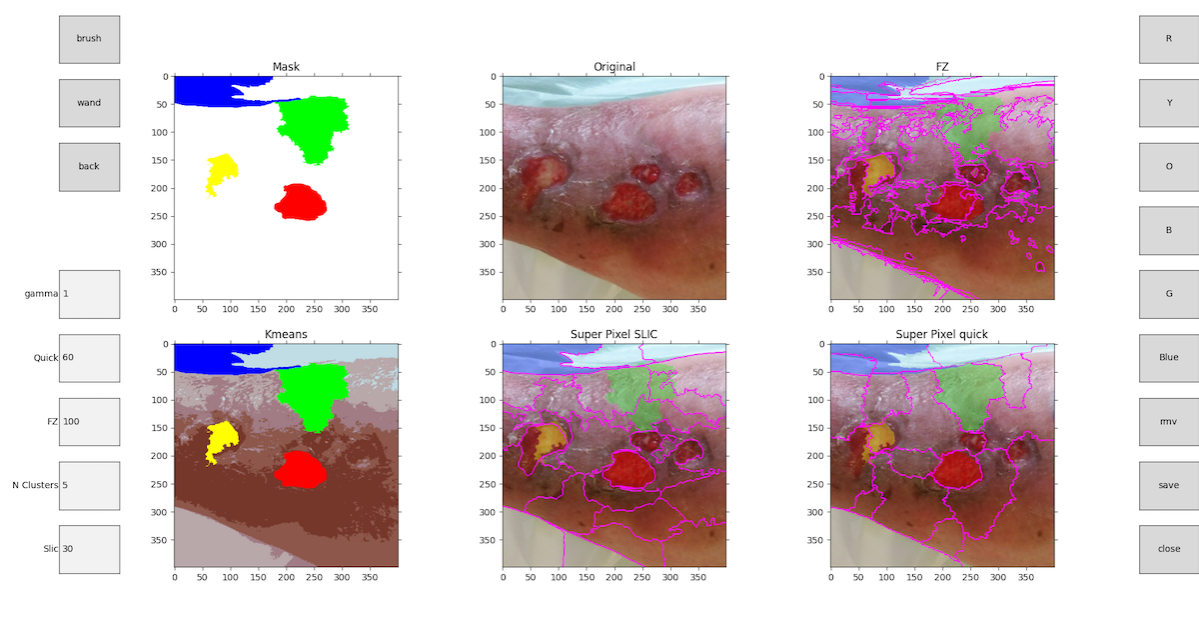
Main menu view. Left options: brush, wand, back, gamma, quick, Felzenszwalb (FZ), N clusters, and simple linear iterative clustering (Slic). Right options: red (R), yellow (Y), orange (O), black (B), gray (G), blue, move (mv), save, and close.

**Figure 5:**
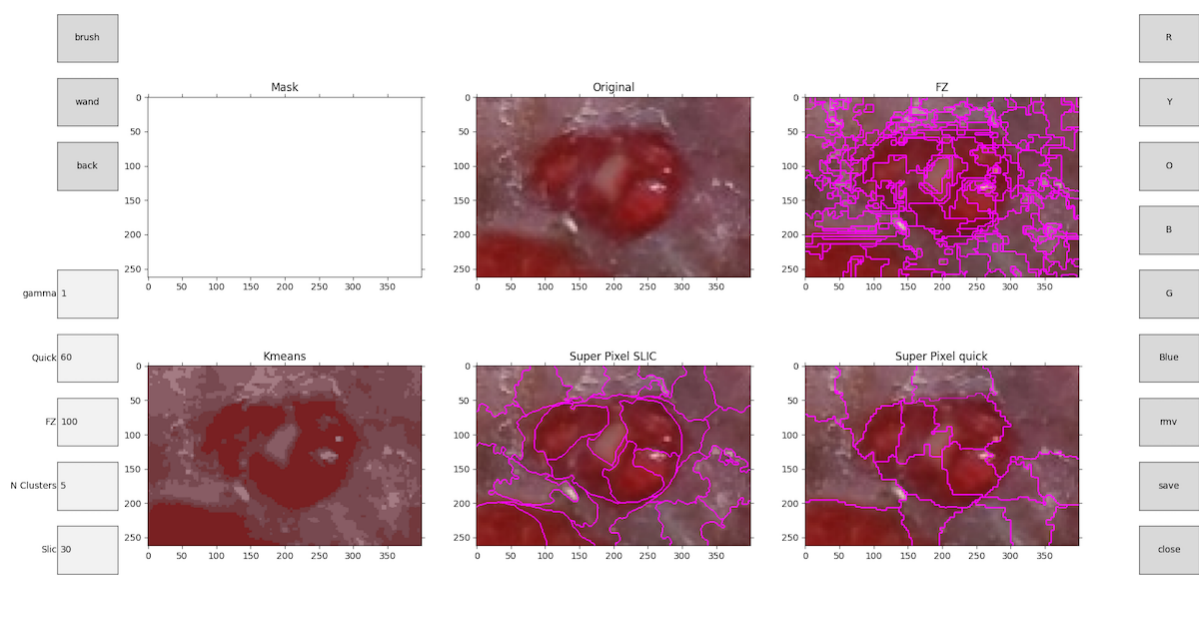
Recalculated partitions from a zoom in the original image. Left options: brush, wand, back, gamma, quick, Felzenszwalb (FZ), N clusters, and simple linear iterative clustering (Slic). Right options: red (R), yellow (Y), orange (O), black (B), gray (G), blue, move (mv), save, and close.

**Figure 6:**
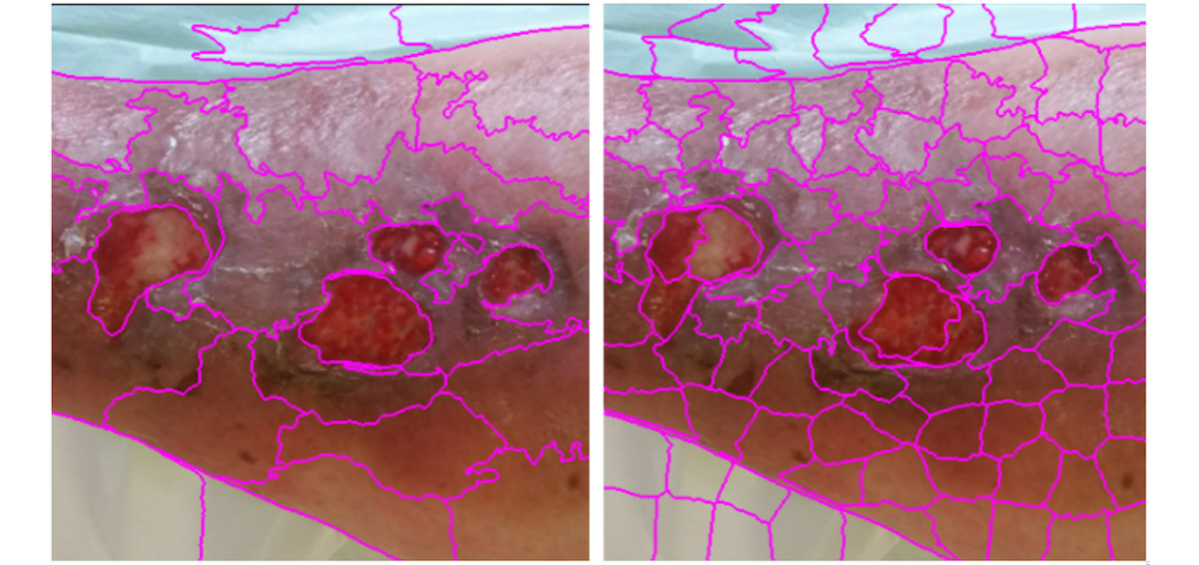
Example of hyperparameters, from left to right: simple linear iterative clustering (SLIC) segmentation with 30 clusters and SLIC segmentation with 100 clusters.

During the first stage, the tool suggests different partitions of the image the user can select based on which segments best suit the labeling objective and define their class (Figure 4). The partitions are calculated automatically, segmenting the image using computer vision methods and separating the different elements. When the user zooms in on parts of the image to be able to increase the precision in complex areas, the segmentation algorithm recalculates over the zoomed section (Figure 5). The user can also change the hyperparameters (parameters whose value is used to control the algorithm) of the segmentation algorithms to recalculate the partitions and get new proposals (Figure 6).

In the second stage, the user will use the segmentations proposed by the tool to select those that best fit the clinical criteria for tissue classification. The user can make use of sections from different proposals. As the user selects the segmentations, the final labeled image will be drawn in the *Mask* section (Figure 4).

Although the proposed tool allows a desired number of tissues to be tagged, this study was based on the hypothesis of labeling 5 types of tissues: intact skin, slough, necrotic, granulated, and background (or no skin). For this reason, only comparisons between these tissue labels will appear in the results presented.

The segmentation process is based on superpixels and clustering methodologies. It uses different configurations of superpixels and clustering to receive different segmentations of the input image. The resulting segmentations are shown to the user to select the partitions that are closest to the tissue distributions.

**Figure 7:**
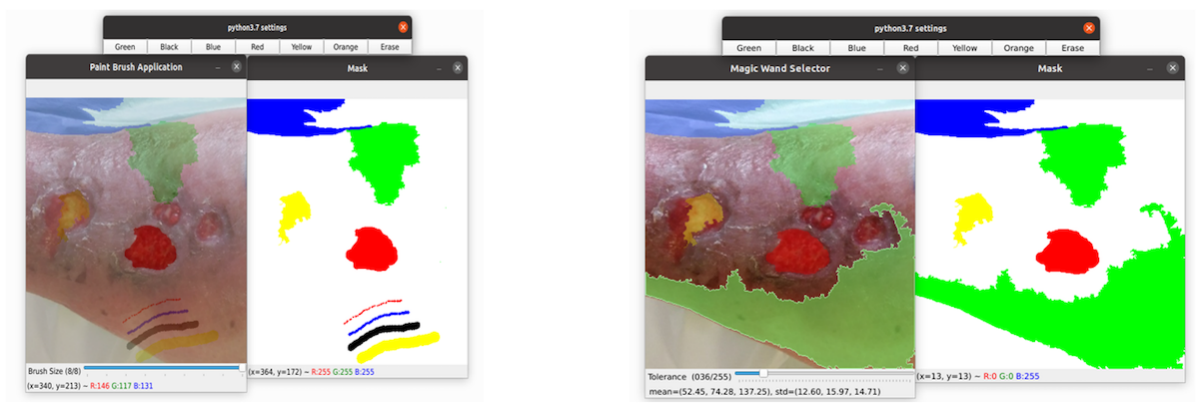
Manual edition tools to classify pixels. RGB: an additive color model with primary colors (red, green, and blue); Std: standard deviation.

In addition, the app has 2 different tools for manual image editing (Figure 7). These tools allow for the correction of mislabeled regions, thus improving the quality of the edges or ambiguous regions hard to segment automatically. The first tool is a brush that allows the user to paint the image using the cursor. The second tool is equivalent to the “magic wand” tool where selecting a pixel in the image causes all the adjacent similar pixels under a threshold to be automatically selected as well.

**Figure 8:**
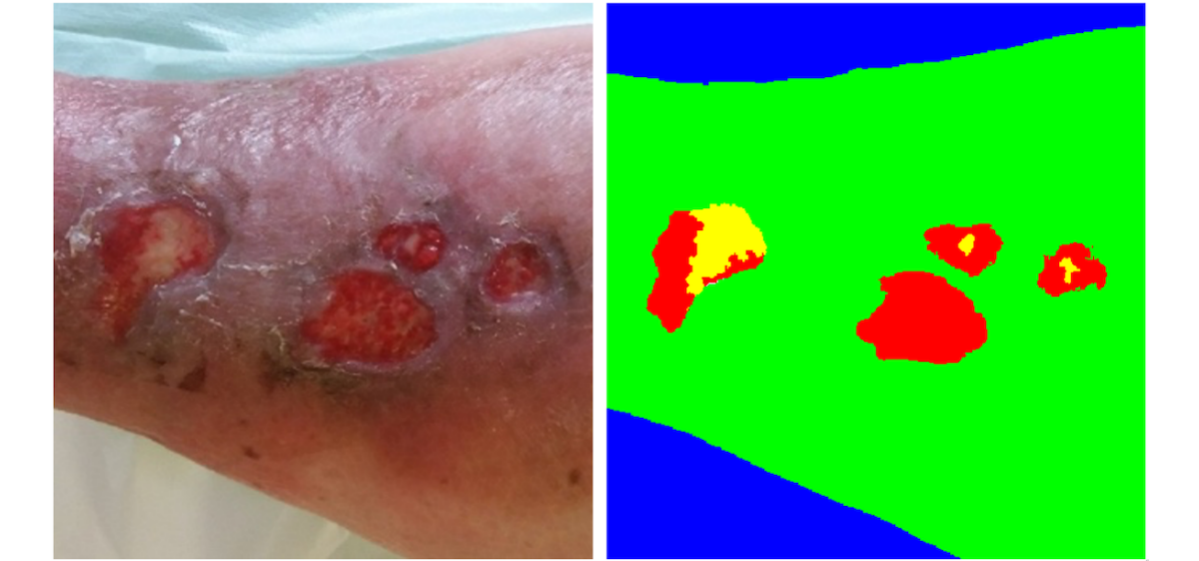
From left to right: original image and labeled image. The classified tissues are intact skin (green), slough (yellow), granulated (red), and background (blue). In this case, there is no presence of necrotic.

At the end of the process, the user can obtain a final labeled image where each pixel value is related to the class of the corresponding pixel in the original image (Figure 8).

As mentioned before, the tool uses different computer visual methods based on superpixels (techniques 1, 2, and 3 below) and clustering (technique 4 below). Superpixels are an aggregation of pixels according to similar characteristics between them, such as raw pixel intensity. There are different algorithms and criteria used to measure the similarity between pixels. Clustering is an unsupervised machine learning technique that involves the grouping of data points in a different number of clusters according to the similarity between them.

#### Technique 1: Felzenszwalb Efficient Graph-Based Segmentation

**Figure 9:**
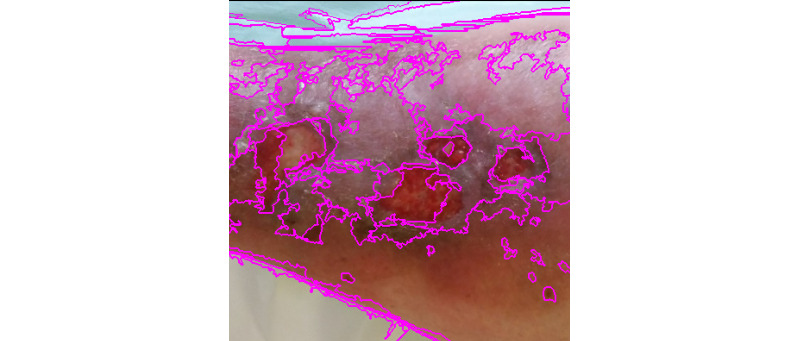
Felzenszwalb segmentation.

Based on superpixels, this technique is a graph-based approach to segmentation [13]. The goal was to develop a computational approach to image segmentation that is broadly useful, much in the way that other low-level techniques such as edge detection are used in a wide range of computer vision tasks. This technique connects elements of the graph according to similarity criteria and a greedy algorithm (Figure 9) to make the boundaries between the different segments more evident.

The similarity criteria used is *Pairwise Region Comparison Predicate*. This predicate is based on measuring the dissimilarity between elements along the boundary of the 2 components. The difference between the 2 components is defined by the minimum weight edge connecting them together.

#### Technique 2: Quickshift Image Segmentation

**Figure 10:**
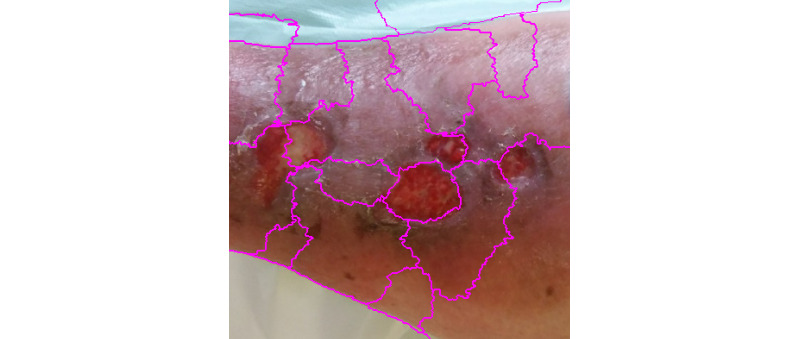
Quickshift segmentation.

This technique uses a “Mean-shift” [14] algorithm that segments an RGB (red, green, and blue primary colors) image (or any image with more than one channel) by identifying clusters of pixels in the joint spatial and color dimensions. Segments are local (superpixels) and can be used as a basis for further processing. The cluster approach is carried out over a 5D space defined by the L,a,b values of the CIELAB (International Commission on Illumination) color space and the x,y pixel coordinates (Figure 10).

Mean-shift is a mode-seeking algorithm that generates image segments by recursively moving to the kernel-smoothed centroid for every data point in the pixel feature space, effectively performing a gradient ascent. The generated segments or superpixels can be large or small based on the input kernel parameters, but there is no direct control over the number, size, or compactness of the resulting superpixels.

#### Technique 3: Simple Linear Iterative Clustering Superpixels

**Figure 11:**
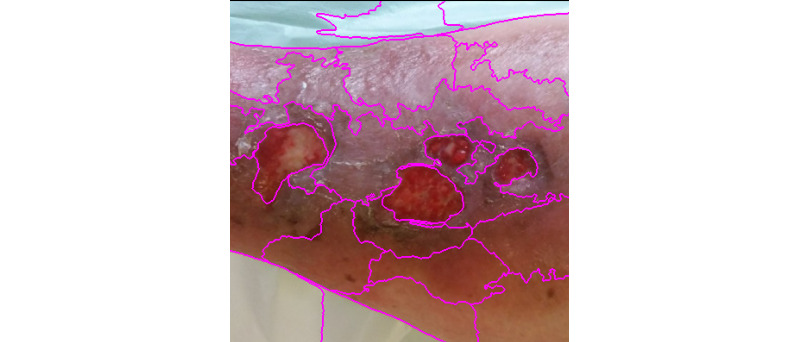
Simple linear iterative clustering (SLIC) segmentation.

This technique’s algorithm [15] consists of simple linear iterative clustering, performing a local clustering of pixels in the 5D space defined by the L,a,b values of the CIELAB color space and the x,y pixel coordinates (Figure 11).

For simple linear iterative clustering, each pixel in the image is associated with the nearest cluster center whose search area overlaps this pixel. After all the pixels are associated with the nearest cluster center, a new center is computed as the average labxy vector of all the pixels belonging to the cluster. We then iteratively repeat the process of associating pixels with the nearest cluster center and recomputing the cluster center until convergence.

#### Technique 4: K-Means Image Segmentation

**Figure 12:**
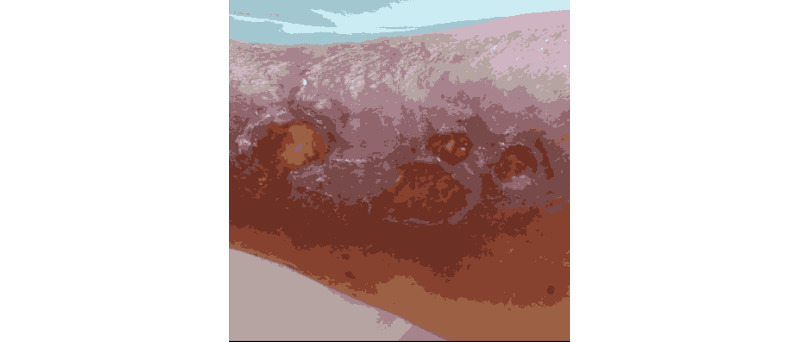
K-means segmentation.

K-means [16] is a clustering method used to divide a set of data into a specific number of groups. For image segmentation, the clusters are calculated by raw pixel intensities. Image pixels are associated to the nearest centroid using Euclidian distance as a similarity measure (Figure 12).

## Results

To evaluate this proposed method, we compared the results obtained by the proposed tool and the results obtained by wound experts using manual segmentation. The manual segmentation was carried out using Gimp, a free cross-platform image editing software, and the experts classified each label pixel by pixel.

Specifically, we compared the time used to classify the wound images in each method and the accuracy of our method against the manual one.

### Time Evaluation

Table 1 shows the time employed to label each one of the data set samples using the gold standard method versus the proposed method. With the proposed method, the image tagging speed is increased by an average of 2.6 times.

**Table 1:** Comparison of the time employed to label each sample of the data set with the 2 referred methods, and the speedup achieved with the proposed method; time notation in minutes and seconds (mm:ss).

Sample	Manual method (time)	New method (time)	Speedup achieved
1	10:30	2:47	3.7x
2	05:35	2:30	2.2x
3	07:30	2:06	3.5x
4	09:15	4:11	2.2x
5	06:30	4:42	1.3x
6	13:24	5:38	2.3x
7	03:54	0:41	5.7x
8	03:02	1:16	2.3x
9	02:44	2:09	1.2x
10	07:06	1:29	4.7x
11	04:20	1:30	2.8x
12	04:42	1:25	3.3x
13	03:05	1:01	3.0x
14	06:37	4:02	1.6x
15	03:21	1:15	2.6x
16	02:49	1:38	1.7x
17	03:18	1:35	2.0x
18	05:07	1:48	2.8x
19	03:59	2:50	1.4x
20	03:17	1:14	2.6x

### Similarity

Precision, recall, and *F*-score measures are used to evaluate the accuracy of labeling algorithms. The image obtained with the gold standard is taken as ground truth. When tagging an image, it is to be expected that the result obtained will be slightly different each time, even if the same tool and the same criteria are used. It is necessary to be able to evaluate whether the samples labeled with the new method are as similar to the gold standard reference samples as would be other samples made with the same method. Therefore, we relabeled all the gold standard samples to compare the quality of the similarity obtained. The exact correlation between gold standard and new labeling method would be 1.0 (Tables 2 and 3).

**Table 2:** Comparison between the gold standard and the proposed labeling method.

Tissue	Precision	Recall	*F*-score
No skin (background)	0.9789	0.9824	0.9804
Intact skin	0.9842	0.9867	0.9854
Granular	0.8426	0.9157	0.8753
Base	0.9309	0.8492	0.8838
Necrotic	0.9871	0.7362	0.8387

**Table 3:** Comparison between the gold standard method samples.

Tissue	Precision	Recall	*F*-score
No skin (background)	0.9919	0.9921	0.9919
Intact skin	0.9938	0.9912	0.9925
Granular	0.8265	0.9377	0.8730
Base	0.9172	0.8821	0.8932
Necrotic	0.9771	0.7622	0.8481

Precision is the relationship between the correctly predicted positive observations and the total expected positive observations. This metric determines how many pixels match out of all the pixels labeled as specific tissue. High precision is related to the low rate of false positives.

Recall, or sensitivity, is the relationship between the correctly predicted positive observations and all positive observations of actual class. This metric determines how many pixels, out of all the pixels that truly matched, were labeled.

*F*-score provides a single score that balances the concerns of both precision and recall in one value. Therefore, this score considers both false positives and false negatives.

## Discussion

### Principal Findings

**Figure 13:**
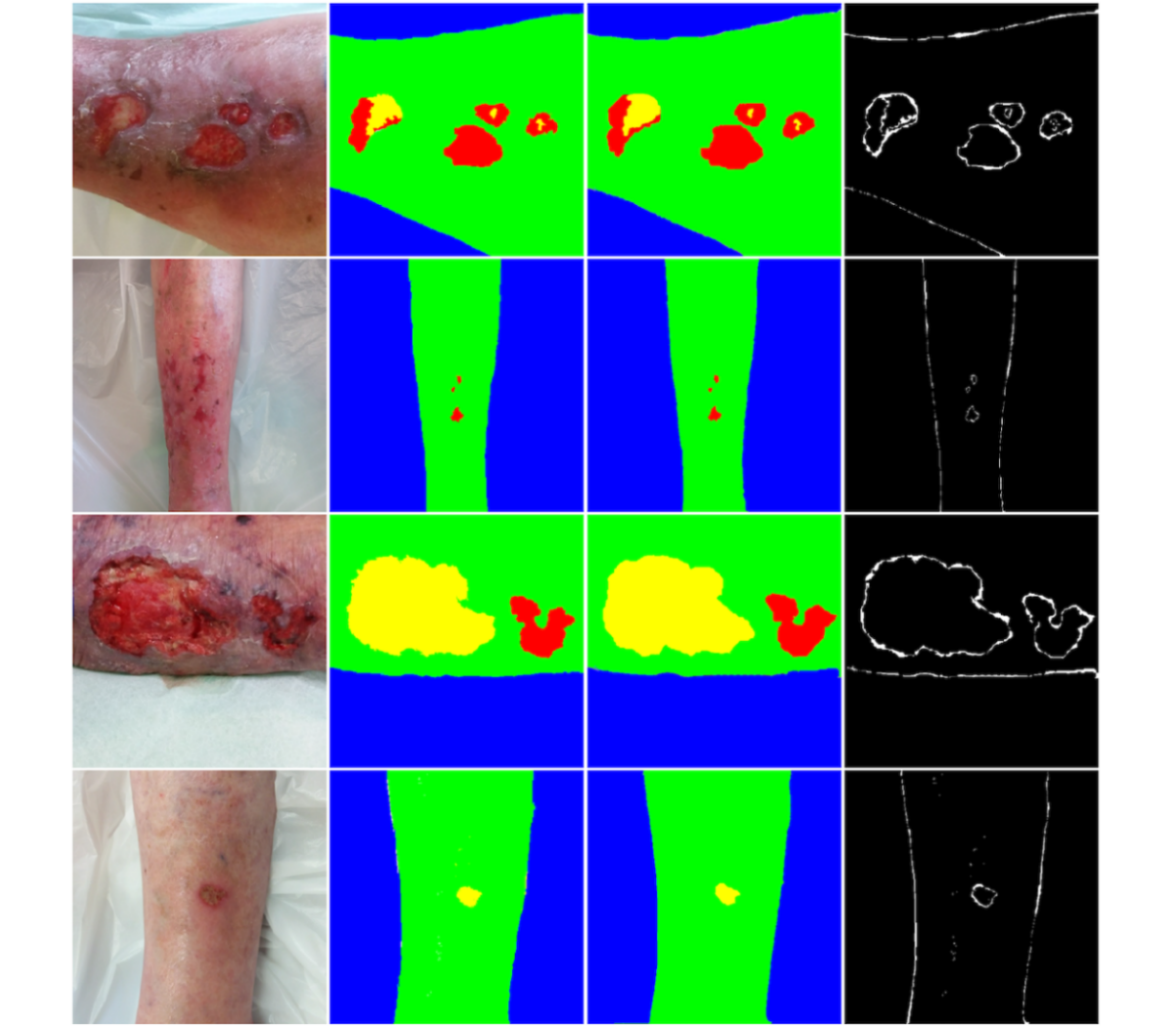
From left to right: examples of original image, labeled image with digital method, labeled with gold standard method, and differences between methods.

By analyzing the difference between images labeled with the 2 methods, we see that the discrepancies are found at the edges of the labeling (Figure 13).

This observation is especially relevant for the evaluation of the smallest elements, where the area or perimeter ratio is more significant and can affect the evaluation of similarity. Likewise, any discrepancy of criteria that may exist in the labeling will affect the minority classes to a greater extent. The majority of the classes (no skin and intact skin) have higher *F*-score values than the rest of the classes.

Evaluating the results in Tables 2 and 3, the results obtained with the 2 methods are highly similar, with almost no difference between the comparison of the labels.

### Conclusions

The proposed method increases tagging speed by an average of 2.6 compared to an advanced image editing user. This gain is larger with untrained users.

The samples obtained with the proposed system are indistinguishable from the samples made with the gold standard.

The incorporation of this type of algorithm will undoubtedly shorten the time required for training a tissue classification network. It provides a tool that can be used by any clinician regardless of their level of knowledge of photo editing. As such, it makes training and using the neural network approach accessible to all in a practical and fast way.

## Abbreviations

CIELAB: International Commission on Illumination
CNN: convolutional neural network
